# Nurses’ knowledge on nosocomial infections preventive measures and its associated factors in Ghana: a cross-sectional study

**DOI:** 10.1186/s12913-023-09942-2

**Published:** 2023-09-01

**Authors:** Samuel Salu, Joshua Okyere, Veronica Okwuchi Charles-Unadike, Mark Kwame Ananga

**Affiliations:** 1 Department of Epidemiology and Biostatistics, University of Health and Allied Sciences, Ho, Ghana; 2 Department of Population and Health, University of Cape Coast, Cape Coast, Ghana; 3 School of Nursing & Midwifery, College of Health Sciences, Kwame Nkrumah University of Science and Technology, Kumasi, Ghana; 4 Department of Population and Behavioral Sciences, University of Health and Allied Sciences, Ho, Ghana

**Keywords:** Knowledge, Nurses, Nosocomial infection, Prevention Measures

## Abstract

**Background:**

Nosocomial infections (NCIs) have been associated with several adverse outcomes including extended hospitalization, persistent disability, heightened antimicrobial resistance, amplified socio-economic disruption, and elevated mortality rates. The adoption of infection prevention strategies has the greatest tendency to significantly reduce the risk and occurrence of NCIs among the population, particularly in resource constrained health systems. This study assessed nurses’ knowledge on NCI preventive measures and its associated factors in Ghana.

**Methods:**

A cross-sectional study was conducted from July to August 2021. A sample of 237 healthcare workers in the Hohoe Municipality was selected to participate in the study. Data was collected with a questionnaire designed in Google Forms and analyzed using Stata version 16.0.

**Results:**

Overall, most of the participants (69.2%) were not knowledgeable about the preventive measures of NCIs. Nurses who were within the age group of 20–40 years [aOR = 0.25 (95% CI = 0.09–0.69), p = 0.007] and 41–60 years [aOR = 0.05 (95% CI = 0.01–0.29), p = 0.001] were significantly less likely to be knowledgeable about the preventive measures of NCIs compared to those who those aged less than 20 years. Nurses who attended in-service training or workshop were approximately 10 times more likely to be knowledgeable about preventive measures of nosocomial infection compared to those who had never attended in-service training or workshop [aOR = 9.55 (95% CI = 1.23–74.36), p = 0.031].

**Conclusion:**

The study concludes that age and participation in-service training or workshop are significant factors that influence the knowledge of healthcare workers in preventive measures for nosocomial infections. These results highlight the importance of providing ongoing training and professional development opportunities to nurses to enhance their knowledge and improve their ability to prevent and control nosocomial infections. Additionally, the study emphasizes the need for targeted training programs that consider the age of nurses, to ensure that training is tailored to their specific needs.

**Supplementary Information:**

The online version contains supplementary material available at 10.1186/s12913-023-09942-2.

## Background

Globally, infections are considered a serious public health concern [1]. While infections occur in different settings including at home, work and in outdoor settings, infections acquired at the healthcare facility pose a significant threat to the overall quality of healthcare delivery. According to the World Health Organisation [2], nosocomial infections (NCIs) or hospital-acquired infections (HAIs) refer to *“an infection occurring in a patient in a hospital or other health care facility in whom the infection was not present or incubating at the time of admission. This includes infections acquired in the hospital but appearing after discharge, and also occupational infections among staff of the facility”*. These infections include urinary tract infections, surgical site infections (e.g., Staphylococcus aureus), bloodstream infections, and lower respiratory tract infections [3].

There are approximately 1.7 million patients worldwide who contract NCIs each year [4]. A systematic review [5] has also estimated NCIs to be increasing worldwide with an annual increasing rate of 0.06, and with the African region having the highest rates of NCIs. Ghana, for instance, has an estimated NCI prevalence of 8.2% [6]. If left unabated, the existence of NCIs would have serious repercussions for health care delivery, time spent at the hospital, and healthcare expenditure. NCIs have been associated with several adverse outcomes including extended hospitalization, persistent disability, heightened antimicrobial resistance, amplified socio-economic disruption, and elevated mortality rates [6–8]. However, the adoption of infection prevention strategies has the greatest tendency to significantly reduce the risk and occurrence of NCI among the population, particularly in resource constrained health systems [9].

Available evidence suggests that adopting NCI prevention involves establishing a protective barrier between vulnerable host and microorganisms [10]. According to the WHO [2], NCI can primarily be prevented either by reducing person-to-person transmission or by preventing transmission from the environment. Reducing person-to-person transmission involves implementing various measures to minimize the spread of infections between patients, healthcare workers, and visitors. This includes promoting proper hand hygiene practices (i.e., handwashing or using hand sanitizers), and practicing safe injection practices [11]. On the other hand, preventing transmission from the environment involves maintaining a clean and hygienic healthcare setting. It encapsulates practices such as sterilization, disinfection of patient equipment, proper waste management and cleaning of the hospital environment [2].

In Ghana, the Ministry of Health and Ghana Health Service has emphasized the importance of adopting preventive measures to control the burden of NCIs [6]. This keen interest in NCI prevention in Ghana is evident in the country’s implementation of a national infection prevention policy and guidelines for healthcare settings [12]. However, it must be noted that having the support of the government agencies and a policy framework is not enough for healthcare workers to implement NCI preventive measures. Their knowledge level is quintessential to the implementation of NCI preventive measures [13]. Nurses’ knowledge on NCI preventive measures is critical for successful implementation and compliance with infection control protocols. Moreover, adequate knowledge has the potential to empower nurses to identify potential risks, implement preventive measures effectively, and respond promptly to infection control challenges [14].

Limited research has been conducted in Ghana to assess the current state of nurses’ knowledge with respect to NCI prevention. While the existing body of literature has examined the extent to which knowledge influences the practice of NCI preventive measures [15, 16], it fails to assess what factors predict the knowledge level of nurses regarding NCI prevention in Ghana. In terms of geographical boundaries, none of the existing studies have investigated the dynamics of nurses’ knowledge on NCI preventive measures in the Volta Region of Ghana. Understanding the level of knowledge and identifying factors associated with nurses’ knowledge gaps can inform targeted interventions and educational programs aimed at improving infection control practices. By addressing these gaps, healthcare facilities in Ghana can enhance their infection prevention and control efforts, leading to a reduction in the burden of NCI and improved patient outcomes. Hence, we aimed to assess nurses’ knowledge on nosocomial infection (NCI) preventive measures and its associated factors in Ghana.

## Methods

### Study design

A cross-sectional study design was employed in this study. This study was carried out in the Hohoe Municipality, in the Volta Region of Ghana. The Hohoe Municipality is one of the eighteen (18) districts in the Volta Region. The city of Hohoe, of which the district was named, serves as the capital and the administrative or local government centre. It shares borders with the Republic of Togo on the east; on the southeast by the Afadzato district and southwest by Kpando Municipality; on the north with Jasikan district; and on the northwest with the Biakoye districts. Its capital, Hohoe, is about 78 km from Ho, the regional capital and 220 km from Accra, the national capital. The Municipality consists of one hundred and two (102) communities with a population of 167,016 projected from the 2010 National Population Census.

### Sample size and sampling procedure

Using Cochrane’s single proportion formula, a sample size of 215 was estimated as follows;

$n=\frac{{z}^{2} p(1-p)}{{d}^{2}}$, considering 5% margin of error, 95% confidence interval = 1.96 and a proportion knowledge of 83.21% from a study conducted by [17]. Where;

n = Estimated sample size.

p = 0.8321.

q= (1-p).

d = margin of error (0.05).

Z = Test Statistic (1.96).

Adding 10% to cater for non-response increased the estimated sample size to 237.

The Hohoe Municipal comprises of four (4) sub-districts and all these sub-districts have health facilities. A simple random sampling was used to select two health facilities from the four (4) sub-districts. This was done by writing the names of all the health facilities from the four (4) sub-districts on pieces of paper, folded for concealment and placed into a container. These papers were thoroughly mixed, and two neutral persons were asked to pick one piece of paper each from the container without opening. In each of the two facilities sampled, nurses who met the inclusion criteria and consented to participate in the study were conveniently selected.

### Inclusion and exclusion criteria

Nurses were selected based on the following inclusion criteria: (1) they must be registered nurses currently working in any of the study sites, (2) they must be present at the time of the survey, and (3) they must express a voluntary interest in participating in the study. Therefore, student nurses and nursing interns were excluded from the study. Additionally, nurses who were on leave during the data collection period were excluded from the study.

### Definition of variables

#### Outcome variable

Knowledge on preventive measures of nosocomial infections was the study’s outcome variable. This was assessed using eleven (11) items on the questionnaire. These items assessed whether respondents (I) have heard about infection prevention (II) could tell if gloves provide complete protection against acquiring or transmitting infections (III) knows if washing hands with soap or an alcohol-based antiseptic decreases the risk of transmission of nosocomial infections (IV) knows if the use of an alcohol-based antiseptic for hand hygiene is as effective as soap and water if hands are not visibly dirty (V) knows if gloves should be worn if blood or body fluid exposure is anticipated or not (VI) knows if there is a need to wash hands before doing procedures that do not involve bodily fluids or not (VII) knows if there is a need to wear the same pair of gloves for multiple patients as long as there is no visible contamination or not (VIII) knows the specific waste disposal buckets according to the level of their contamination (VIV) knows the written formula for preparing 0.5% chlorine solution (X) knows how long instrument or equipment should be disinfected and (XI) knows disease that are transmitted by needle stick injury.

For each of the items (I-X), respondents were asked to choose from the two responses “yes” or “no” provided. For question (XI), respondents were provided with a list of diseases (HBV, HCV, TB, HIV) to choose from. Respondents were allowed to choose from the list multiple times. A composite knowledge score was obtained by assigning a score of 1 to all the positive responses to the eleven (11) questions. All negative responses on the other hand were assigned the score of 0. A mean score was generated by adding all these responses and nurses who scored below the mean were considered “not knowledgeable” on the preventive measures of nosocomial infections, whereas nurses who scored above the mean were considered “knowledgeable”. The study incorporated the classification of knowledge on the preventive measures of nosocomial infections into “not knowledgeable” and “knowledgeable” from previous studies [18–20].

#### Explanatory variable

For the study, five (5) explanatory variables were considered in our estimations. These variables included age, sex, level of education, years of working experience and in-service training or workshop. None of these variables were chosen at random; rather, they were chosen based on the findings of previous studies on knowledge in preventive measures of nosocomial infections among healthcare workers [21–23]. In assessing these socio-demographic information of the respondents, age and years of working experience were collected as continuous variables and categorized into (< 20 years, 20–40 years, 41–60 years) and (1–10 years, 11–20 years and > 20 years) respectively. Respondents’ sex (male or female), level of education (certificate, diploma, degree, masters or doctoral), and in-service training or workshop (yes or no) were collected as categorical variables.

### Data collection

The study covered a period from July 2021 to August 2021. Nurses working in the Hohoe Municipality and whose consents are been sought during the time of the study were recruited to participate in the study. Data was collected using a well-structured questionnaire comprising of both open and close-ended questions which were pretested. The questionnaire was designed in Google Forms. It comprised of 35 items related to the socio-demographic factors, knowledge in preventive measures and practice of preventive measures of NCIs.

### Data analysis

The data was extracted from Google Forms to Excel Sheet for cleaning and then exported into STATA V.16.0 (StataCorp. 2019. Stata Statistical Software: Release 16. College Station, TX: StataCorp LLC.) analysis. To ensure the quality of the data extracted, double entry was done to address discrepancies which may have occurred during data extraction. The data was extensively cleaned again in STATA V. 16.0 (StataCorp. 2019. Stata Statistical Software: Release 16. College Station, TX: StataCorp LLC.) before analysis was carried out. Descriptive statistics were performed to interpret the socio-demographic features including age, sex, level of education, work experience, and in-service training or workshop on nosocomial infection prevention. However, inferential statistics were done to test the association between socio-demographic factors and healthcare workers’ knowledge in preventive measures of nosocomial infections. Frequencies and percentages related to the study findings were presented using tables and graphs.

## Results

Table 1 provides a distribution of the socio-demographic characteristics of the respondents. The majority of the healthcare workers were females 124 (52.3%) while males were 113 (47.7%). More than two-thirds of them, 195 (82.7%) were within the age group of (20–40) years. Most of the participants, 127 (53.6%) were Diploma holders. Regarding their work experience, almost all of them 204 (86.1%) had work experience ranging between (1–10) years. Out of the total 237 healthcare workers, 218 (92.0%) had attended in-service training or workshop on nosocomial infection prevention while 19 (8.0%) had never attended any in-service training or workshop on nosocomial infection prevention.

**Table 1:** Distribution of the socio-demographic characteristics of the respondents

Variable	Frequency (n = 237)	Percentage (%)
**Sex**		
Female	124	52.3
Male	113	47.7
**Age**		
< 20 years	19	8.0
20–40 years	195	82.3
41–60 years	23	9.7
**Level of Education**		
Certificate	15	6.3
Diploma	127	56
Degree	80	33.8
Masters	14	5.9
Doctoral	1	0.4
**Years of Working Experience**		
1–10 years	204	86.1
11–20 years	23	9.7
> 20 years	10	4.2
**In-service training or Workshop**		
No	19	8.0
Yes	218	92.0

### Knowledge of nurses on preventive measures of nosocomial infections

Table 2 shows the distribution of nurses’ knowledge on NCI preventive measures. Out of the total 237 nurses who participated in the study, more than 90% 216 (91.1%) indicated that they had heard about infection prevention. More than half 148 (68.5%) out of the 216 (91.1%) who had heard about infection prevention believed that gloves cannot provide complete protection against acquiring or transmitting infections. Almost all of them 213 (98.6%) believed that washing your hands with soap or using an alcohol-based antiseptic decreases the risk of transmission of NCI. More than 80% of them 187 (86.6%) also indicated that the use of an alcohol-based antiseptic for hand hygiene is as effective as soap and water if hands are not visibly dirty. All of them 216 (100%) agreed that there is a need to wash hands before doing procedures that do not involve bodily fluids. Furthermore, 198 (83.5%) of the nurses know the specific waste disposal buckets according to the level of their contamination. Most of the participants, 210 (88.6%), indicated that instruments or equipment should be disinfected for 10 min.

**Table 2:** Distribution of nurses’ knowledge on preventive measures of nosocomial infections

Variables	Frequency (n = 237)	Percentage (%)
**Heard about infection prevention**		
No	21	8.9
Yes	216	91.1
**Gloves provide complete protection against acquiring or transmitting infections (n = 216)**		
No	148	68.5
Yes	68	31.5
**Hand washing with soap and water or an alcohol-based antiseptic decreases the risk of nosocomial infection transmission (n = 216)**		
No	3	1.4
Yes	213	98.6
**Using an alcohol-based antiseptic for hand hygiene is as effective as using soap and water if hands are not visibly dirty (n = 216)**		
No	29	13.4
Yes	187	86.6
**Gloves should be worn if blood or body fluid exposure is anticipated (n = 216)**		
No	38	17.6
Yes	178	82.4
**There is a need to wash hands before carrying out procedures that do not involve bodily fluids (n = 216)**		
No	0	0.00
Yes	216	100
**There is a need to wear the same pair of gloves for multiple patients as long as there is no visible contamination (n = 216)**		
No	201	93.1
Yes	15	6.9
**Know the specific waste disposal buckets according to the level of their contamination**		
No	39	16.5
Yes	198	83.5
**Know the written formula for preparing 0.5% chlorine solution**		
No	51	21.5
Yes	186	78.5
**Duration for the disinfection of instruments or equipment**		
10 min	210	88.6
1 h	20	8.4
24 h	7	3.0
**Diseases transmitted by needle stick injury (More than one answer is possible)**		
HBV; HCV	1	0.4
HBV; HCV; HIV	117	49.4
HBV; HCV; TB; HIV	3	1.3
HBV; HIV	52	21.9
HBV; TB; HIV	2	0.8
HIV	58	24.5
TB; HIV	4	1.7

***HBV*: Hepatitis B Virus;** ***HCV*: Hepatitis C Virus;** ***HIV*: Human Immunodeficiency Virus;** ***TB*: Tuberculosis**

**Fig. 1:**
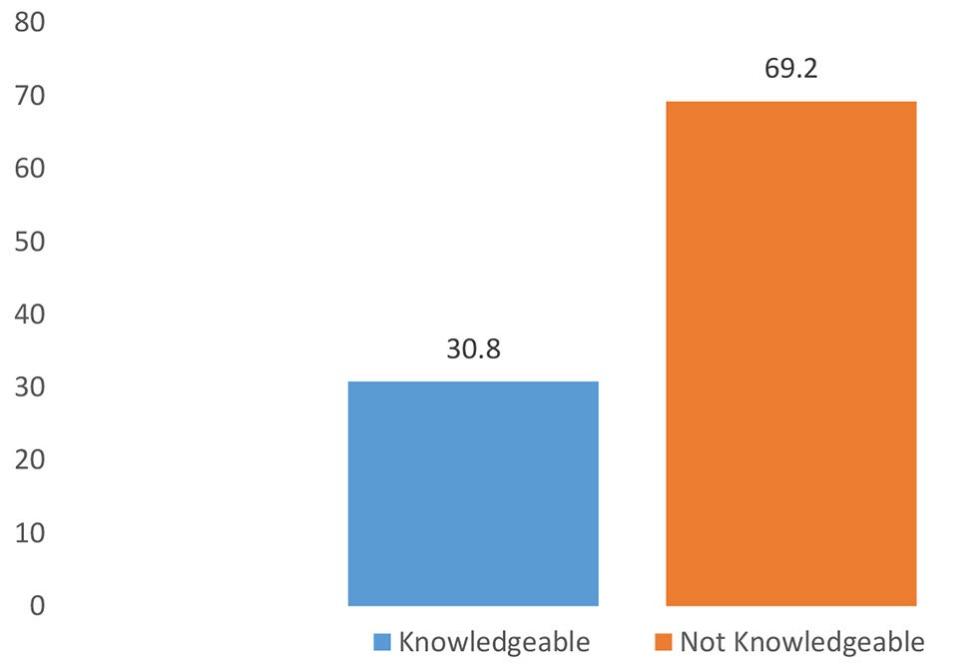
Overall level of knowledge of healthcare workers in preventive measures of nosocomial infections

A knowledge mean score was generated using the items used to measure the level of knowledge among the nurses. Those who scored below the mean were considered not knowledgeable on the preventive measures of NCIs, whereas those who scored above the mean were considered knowledgeable. The findings from this study revealed that 164 (69.2%) of the participants were not knowledgeable on the preventive measures of NCIs (Fig. 1).

### Factors associated with nurses’ knowledge on preventive measures of nosocomial infections

Age and in-service training or workshop were the only factors that were significantly associated with the participants’ knowledge on NCI preventive measures. Sex, level of education and years of working experience showed no significant association with nurses’ knowledge on NCI preventive measures. Specifically, nurses who were within the age group of 20–40 years [aOR = 0.25 (95% CI = 0.09–0.69), p = 0.007] and 41–60 years [aOR = 0.05 (95% CI = 0.01–0.29), p = 0.001] were significantly less likely to be knowledgeable about the preventive measures of NCIs compared to those who those aged less than 20 years. Nurses who attended in-service training or workshop were approximately 10 times more likely to be knowledgeable about preventive measures of nosocomial infection compared to those who had never attended in-service training or workshop [aOR = 9.55 (95% CI = 1.23–74.36), p = 0.031] (Table 3).

**Table 3:** Factors associated with nurses’ knowledge on preventive measures of NCIs

Variable	Level of Knowledge in Preventive Measure		cOR [95% CI]	P-values	aOR [95%CI]	P-values
	Not Knowledgeable = 164 n(69.20%)	Very Knowledgeable = 73 n(30.80%)				
**Sex**						
Female	92 (56.10)	32 (43.84)	0.51 [0.12–0.71]	0.081	-	-
Male	72 (43.90)	41 (56.16)	6.12 [1.01–1.99]	0.132	-	-
**Age**						
< 20 years	7 (4.27)	12 (16.44)	**Ref**		**Ref**	
20–40 years	136 (82.93)	59 (80.82)	**0.25 [0.09–0.67]**	0.006*	**0.25 [0.09–0.69]**	0.007*
41–60 years	21 (12.80)	2 (2.74)	**0.06 [0.01–0.31]**	0.001***	**0.05 [0.01–0.29]**	0.001***
**Level of Education**						
Certificate	10 (6.10)	5 (6.85)	6.43 [2.62–9.67]	0.072	-	-
Diploma	84 (51.22)	43 (58.90)	0.87 [1.08–6.95]	0.111	-	-
Degree	56 (34.15)	24 (32.88)	5.43 [0.03–1.72]	0.065	-	-
Masters	13 (7.93)	1 (1.37)	0.97 [0.07–0.49]	0.232	-	-
Doctoral	1 (0.60)	0 (0.00)	3.25 [1.33–4.11]	0.142	-	-
**Years of Working Experience**						
1–10 years	136 (82.92)	68 (93.15)	5.16 [2.04–6.63]	0.074	-	-
11–20 years	18 (10.98)	5 (6.85)	2.95 [0.19–1.42]	0.081	-	-
> 20 years	10 (6.10)	0 (0.00)	1.66 [0.01–0.31]	0.061	-	-
**In-service training or Workshop**						
No	18 (10.98)	1(1.37)	**Ref**		**Ref**	
Yes	146(89.02)	72(98.63)	**8.88 [1.16–67.81]**	0.035*	**9.55 [1.23–74.36]**	0.031*

*aOR*: adjusted odds ratio, *CI*: confidence interval, c*OR*: crude odds ratio, *Ref*: Reference point; *p < 0.05; ***p < 0.001

## Discussion

Recognizing the importance of NCI prevention to the healthcare system of Ghana [6, 12], we assessed nurses’ knowledge on NCI preventive measures and its associated factors in Ghana. Our study revealed that more than two-thirds of nurses (69.2%) were not knowledgeable about NCI preventive measures. The observed proportion of nurses who were knowledge about NCI prevention measures is less when compared a previous study conducted in Ethiopia [10] where 90% of nurses had good knowledge on NCI preventive measures. Nevertheless, our findings align with a prior study conducted in Tamale, Ghana [21] which revealed that only 50% of nurses were knowledgeable about infection prevention measures, including NCIs. The observed low knowledge on NCI preventive measures among nurses poses a significant threat to patient safety. This is in the sense that patients often rely on nurses to provide safe and effective care. Therefore, having a low knowledge about NCI preventive measures implies that nurses may be involved in practices that exacerbate the risk of NCI transmission [13, 15]. Our findings, thus, underscore a need for the Ministry of Health, Ghana Health Service and hospital administrators to intensify education and sensitization initiatives to improve nurses’ knowledge regarding NCI preventive measures.

The study revealed that having participated in an in-service training or workshop was positively associated with nurses’ knowledge on NCI preventive measures. That is, the likelihood of being knowledgeable about NCI preventive measures was significantly higher among those who had participated in an in-service training or workshop compared to those who had not participated in such initiatives. Similar findings have been reported in North-East Ethiopia [23] and Nigeria [25]. A plausible explanation for this result could be that in-service training and workshops serve as reinforcement mechanisms for existing knowledge. Even if nurses have received prior education on infection control, attending training sessions provides an opportunity to refresh their knowledge, identify areas for improvement, and correct any misconceptions or outdated practices. The repetition of key concepts and information during the training sessions has the potential to reinforce the importance of NCI prevention and increases retention of knowledge among nurses [25].

Another finding from this study was the significant association between age and nurses’ knowledge on NCI prevention. Contrary to previous studies that have shown that nurses’ knowledge on NCI prevention increases with increasing age [22–24], we found that older age was associated with lower odds of being knowledgeable about NCI prevention compared to those of younger age. That is, the present study challenges the existing literature that posits that older nurses tend to be more knowledgeable about NCI prevention through years of experience and working collaboratively with senior staff [22]. It is possible that younger nurses, who may have recently completed their education or training, are likely to have been exposed to more up-to-date information and guidelines regarding NCI prevention. They may have received more comprehensive training that includes the latest research, technological advancements, and evidence-based practices. In contrast, older nurses may not have had the same exposure to these updated resources, leading to a knowledge gap between the age groups. We also postulate that older nurses may be less inclined to adopt new practices or update their knowledge base, especially if they have been practicing for a long time without encountering significant issues related to NCI. This resistance to change can result in a slower uptake of new information and guidelines, hence, explaining their lower knowledge levels regarding NCI prevention.

### Implications for policy and practice

The study highlights the urgent need for the Ministry of Health, Ghana Health Service, and hospital administrators to prioritize education and sensitization initiatives on NCI preventive measures for nurses. Also, the positive association between participation in in-service training or workshops and nurses’ knowledge on NCI preventive measures emphasizes the importance of these initiatives. Healthcare institutions should provide regular opportunities for nurses to attend such training sessions, as they serve as reinforcement mechanisms for existing knowledge and contribute to improved understanding and implementation of NCI prevention measures. To bridge the knowledge gap observed among older nurses, healthcare institutions should design and implement tailored training programs that specifically address their needs. These programs should focus on updating their knowledge base, addressing resistance to change, and providing them with the necessary skills to adopt current NCI preventive measures.

### Strengths and limitations

The strength of this study lies in the use of appropriate methodology to estimate the sample and analyze the data. Nevertheless, there are some limitations that must be taken into consideration. As the study relied on a cross-sectional design, it is not possible to establish a causal pathway between the age and in-service training as determinants of nurses’ knowledge on preventive measure for NCIs. The quantitative approach to this study does not provide an in-depth insight into other underlying factors that might influence the observed associations. Therefore, there is a need for qualitative research to gain a more comprehensive and nuanced understanding of nurses’ knowledge on preventive measures for NCIs. Another limitation of this study is that it focused only on nurses. Hence, the findings may not reflect the current knowledge of other healthcare workers including general medical practitioners, surgeons, and laboratory technicians.

## Conclusion

A significant proportion of nurses in Ghana lack knowledge on NCI prevention. The study concludes that age and participation in-service training or workshop are significant factors associated with nurses’ knowledge NCI prevention. These results highlight the importance of providing ongoing training and professional development opportunities to nurses to enhance their knowledge and improve their ability to prevent and control nosocomial infections. Additionally, the study emphasizes the need for targeted training programs that consider the age of nurses, to ensure that training is tailored to their specific needs and learning styles.

### Electronic supplementary material

Below is the link to the electronic supplementary material.

Supplementary Material 1

## Data Availability

All data generated or analyzed during this study are included in this published article.

## Abbreviations

aOR: Adjusted odds ratio
cOR: Crude odds ratio
HAIs: Hospital Acquired Infections
HIV: Human Immunodeficiency virus
HBV: Hepatitis B Virus
HCV: Hepatitis C Virus
IPC: Infection prevention and Control
ICU: Intensive Care Unit
NCI: Nosocomial infection
NCIs: Nosocomial infections
WHO: World Health Organization
