# Supplementary material for: Nurses’ knowledge on nosocomial infections preventive measures and its associated factors in Ghana: a cross-sectional study
Source: BMC Health Serv Res. 2023 Sep 1;23:941. doi: 10.1186/s12913-023-09942-2 (PMC10474753; doi:10.1186/s12913-023-09942-2)
Supplement: Supplementary file 1 — Supplementary Material 1 [file 12913_2023_9942_MOESM1_ESM.pdf]

QUESTIONNAIRE

SECTION 1: SOCIO-DEMOGRAPHIC CHARACTERISTICS

1. Sex: Male ☐ Female ☐

2. Age: …………………….

3. What is your highest level of education?

DIPLOMA ☐ DEGREE ☐ MASTERS ☐

Other (*please specify*) ………………………………………………

4. Year of working experience…………………

5. Have you ever participated in any in-service training or workshop about nosocomial infection prevention or infection control? YES ☐ NO ☐

SECTION 2: LEVEL OF KNOWLEDGE OF NOSOCOMIAL INFECTIONS

To complete this section, please circle the number which corresponds to how you agree with the given statement.

1. Strongly disagree 2. Disagree 3. Agree 4. Strongly agree

**6**	I am fully aware of nosocomial infections	**1**	**2**	**3**	**4**
**7**	Nosocomial infections are also known as hospital acquired infections	**1**	**2**	**3**	**4**
**8**	Nosocomial infections occur during 48 to 72 hours after admission or during a specified period of 3 to 10 days after dismissal	**1**	**2**	**3**	**4**
**9**	Nosocomial infections may be transmitted via medical equipment such as syringes, thermometers, needles, catheters, stethoscopes etc.	**1**	**2**	**3**	**4**

**10**	Nosocomial infections can be transmitted from one person to another	**1**	**2**	**3**	**4**
**11**	Transmission of healthcare-associated pathogens takes place through direct and indirect contact, droplets, air, and a common vehicle.	**1**	**2**	**3**	**4**
**12**	Healthcare workers are immune to nosocomial infections	**1**	**2**	**3**	**4**
**13**	Invasive procedures increase the risk of nosocomial infections	**1**	**2**	**3**	**4**

SECTION 3: LEVEL OF KNOWLEDGE IN PREVENTIVE MEASURES OF NOSOCOMIAL INFECTION

**14**	Did you hear about infection prevention?	1. Yes<br>2. No	
**15**	Can gloves provide complete protection against acquiring or transmitting infections?	1. Yes<br>2. No	
**16**	Can washing your hands with soap or an alcohol-based antiseptic decreases the risk of transmission of nosocomial infections?	1. Yes<br>2. No	
**17**	Is the use of an alcohol-based antiseptic for hand hygiene as effective as soap and water if hands are not visibly dirty?	1. Yes<br>2. No	
**18**	Should gloves be worn if blood or body fluid exposure is anticipated?	1. Yes<br>2. No	
**19**	Is there a need to wash hands before doing procedures that do not involve bodily fluids?	1. Yes<br>2. No	
**20**	Is there a need to wear the same pair of gloves for multiple patients as long as there is no visible contamination?	1. Yes<br>2. No	

**21**	Do you know specific waste disposal buckets according to the level of their contamination?	1. Yes<br>2. No	
**22**	Do you know the written formula for preparing 0.5% chlorine solution?	1. Yes<br>2. No	
**23**	For how long should instrument or equipment be disinfected?	1. 10 minutes<br>2. 1hr<br>3. 24hrs<br>4. Other (*specify*)	
**24**	Which of the following disease is transmitted by needle stick injury? (More than one answer is possible)	1. HBV<br>2. HCV<br>3. TB<br>4. HIV<br>5.Other (*specify*)	

THANK YOU FOR PARTICIPATING!
